# Hair cortisol-to-cortisone ratio and dental caries in adolescents: a cross-sectional analysis of glucocorticoid imbalance and caries prevalence

**DOI:** 10.1186/s12903-026-08785-7

**Published:** 2026-06-05

**Authors:** Anara Zhumadilova, Bakytgul Yermekbayeva, Alibek Kossumov, Almagul Kushugulova, Samat Kozhakhmetov, Maria Modayil, Khyathi Gadag, Graciela Muniz-Terrera, Zelalem T. Haile, Adil Supiyev

**Affiliations:** 1 Ohio University, Athens, OH USA; 2 National Laboratory Astana, Center for Life Sciences, Nazarbayev University, Astana, Kazakhstan

**Keywords:** Cortisol-to-cortisone ratio, Dental caries, DMFT, Adolescents, Chronic stress biomarkers, Glucocorticoids

## Abstract

**Background:**

Chronic stress and dysregulation of the hypothalamic-pituitary-adrenal (HPA) axis may influence oral health, yet evidence linking long-term glucocorticoid exposure to dental caries in adolescents remains limited. Hair cortisol and cortisone provide integrated biomarkers of chronic HPA-axis activity, and their ratio reflects glucocorticoid metabolic balance and 11β-hydroxysteroid dehydrogenase activity, which may be influenced by chronic stress-related physiological dysregulation. The objective was to examine the association between hair cortisol, cortisone, and their ratio with dental caries prevalence and severity in adolescents.

**Methods:**

This secondary cross-sectional analysis included 550 adolescents aged 14–15 years from the Kazakhstan Adolescent Health Study. Dental caries was assessed using the DMFT index. Hair glucocorticoid concentrations were measured by liquid chromatography-mass spectrometry, log-transformed, and the cortisol-to-cortisone ratio (CCR) was standardized (z-score). Multivariable logistic regression estimated odds ratios across hierarchical models adjusting for sociodemographic, socioeconomic, and behavioral factors, with school region as a fixed effect and robust standard errors. Zero-inflated negative binomial (ZINB) models examined associations with caries count.

**Results:**

Among 550 participants, 84% had DMFT ≥ 1. Although unadjusted analyses did not show significant differences in CCR by caries status (*p* = 0.079), higher standardized CCR was associated with increased odds of dental caries after multivariable adjustment (OR = 1.40, 95% CI: 1.04–1.89 per SD increase). Results were consistent across sensitivity analyses, including Firth’s penalized logistic regression (OR = 1.36, 95% CI: 1.01–1.85). In ZINB analyses, CCR was not significantly associated with caries count. Individual glucocorticoids showed weaker, less consistent associations than their ratio.

**Conclusions:**

In this cross-sectional sample, higher CCR was associated with greater odds of dental caries after adjustment for measured covariates. Although elevated CCR may reflect physiological dysregulation associated with chronic stress, it should not be interpreted as a direct measure of stress. These findings are exploratory, require cautious interpretation given the cross-sectional design and absence of a significant unadjusted association, and need confirmation in longitudinal studies.

**Supplementary Information:**

The online version contains supplementary material available at 10.1186/s12903-026-08785-7.

## Background

Chronic stress has emerged as an important determinant of oral health through both biological and behavioral mechanisms [1]. Stress activates the HPA axis, leading to the secretion of glucocorticoids, primarily cortisol and its inactive metabolite cortisone, which regulate metabolism, immune function, and inflammatory responses [2]. Alterations in cortisol-cortisone metabolism mediated by 11β-hydroxysteroid dehydrogenase (11β-HSD) enzymes may impair salivary antimicrobial function, alter oral microbial ecology, and reduce buffering capacity, biological factors that have been associated with caries progression to the dentine level [3].

Dental caries remains the most prevalent chronic disease among adolescents worldwide and represents a major public health concern due to its long-term consequences for quality of life, academic performance, and overall well-being [4, 5]. In Kazakhstan, the burden of untreated dental caries among adolescents aged 12–15 years remains extremely high and has shown little improvement over the past decade, particularly in the northern and eastern regions of the country, which are characterized by greater socioeconomic disadvantage and more unfavorable dietary and oral hygiene behaviors [6]. However, despite improvements in access to preventive dental services, the biological and psychosocial pathways that contribute to caries development in this population remain insufficiently understood [4, 6, 7]. Traditional measures of stress, such as salivary cortisol, reflect short-term physiological fluctuations and are highly sensitive to momentary conditions [5, 8]. In contrast, hair cortisol and hair cortisone provide cumulative measures of long-term stress exposure over several weeks or months [9]. These biomarkers have been validated as reliable indicators of chronic HPA-axis activity [10] and are increasingly used to assess long-term in adolescent populations [9, 11, 12].

Recent studies have reported associations between elevated hair cortisol concentrations and increased dental caries among adolescents and young adults [13]. Additionally, research conducted in the United States, Europe, and Asia has identified socioeconomic and family-related stress as important determinants of elevated cortisol levels and increased caries risk [14–16]. Behavioral mechanisms, such as stress-related consumption of sugary foods and reduced oral hygiene, may further contribute to caries development [17–19]. However, relatively few studies have simultaneously examined hair cortisol, hair cortisone, and the CCR, a marker reflecting 11β-hydroxysteroid dehydrogenase activity and glucocorticoid metabolic balance [13, 20]. Although elevated CCR may reflect physiological dysregulation associated with chronic stress exposure, it should not be interpreted as a direct measure of psychological stress itself. Moreover, no studies to date have investigated these associations in Kazakhstan, where adolescent caries prevalence remains high and biological pathways linking chronic stress to oral health outcomes remain underexplored [6, 21]. To our knowledge, this is the first study to simultaneously examine hair cortisol, hair cortisone, and the CCR in relation to dental caries among adolescents in Central Asia [13].

Therefore, the aim of this study was to examine the association between chronic stress biomarkers, including hair cortisol, hair cortisone, and the CCR, and the presence of dental caries among adolescents aged 14–15 years in Kazakhstan. We hypothesized that higher levels of these glucocorticoid biomarkers, reflecting chronic HPA-axis activity and metabolic imbalance, would be associated with a greater likelihood of dental caries.

## Methods

### Study design and data source

This study is a secondary analysis of data from the Kazakhstan Adolescent Health Study (KAHS), a cross-sectional survey conducted between 2014 and 2015 by the Laboratory of Epidemiology and Public Health at the National Laboratory Astana, Nazarbayev University, in collaboration with University College London, United Kingdom [6]. The KAHS protocol was primarily based on the Health Behavior in School-aged Children (HBSC) study [22], a World Health Organization (WHO) collaborative cross-national survey conducted in 44 countries to examine adolescents’ health and well-being within their social contexts. The KAHS survey adapted the HBSC protocol following agreement with the HBSC steering committee. For the dental health component, elements of the Children’s Dental Health Survey (CDHS) protocol were incorporated into structured questionnaires for both schoolchildren and their parents [23].

The target population comprised children aged 11–15 years residing in five regions of Kazakhstan: Astana (capital city), Kokshetau (northern Kazakhstan), Oskemen (eastern Kazakhstan), Semey (eastern Kazakhstan), and Qaragandy (central Kazakhstan). These regions were selected based on the geographic distribution of Nazarbayev Intellectual Schools (NIS), as the original study aimed to assess the overall health of NIS students across different parts of the country [6]. Qaragandy was excluded from the present analysis because dental examination data were not available for that region. In each region, two schools representing different educational settings were selected: a Nazarbayev Intellectual School (NIS), a government-funded institution designed for academically talented students, and a regular public school serving the general population. Both school types provide free education and do not require entry fees.

Parents of eligible adolescents were invited through parent-teacher meetings organized at participating schools. They received written and verbal information about the study and provided informed consent for their child’s participation. Structured questionnaires collected information on sociodemographic characteristics, socioeconomic conditions, health behaviors, and environmental factors. Objective measurements obtained by trained staff included height, weight, waist and hip circumferences, blood pressure, spirometry, buccal epithelium swabs, hair samples, and dental examination.

## Study population

The present analysis included adolescents aged 14–15 years with complete data on dental examination and hair glucocorticoid measurements. Participants were drawn from the original KAHS sample of 2,149 adolescents aged 11–15 years (response rate 68%). A total of 550 participants met the inclusion criteria for the current study. Adolescents aged 14–15 years were selected for this particular study from the larger sample. This developmental stage is widely used in dental epidemiology because of the near-complete eruption of permanent dentition, which therefore allows for reliable assessment of cumulative caries experience [24, 25]. In addition, mid-adolescence is a critical period during which behavioral exposures and biological stress responses become established, enabling investigation of the relationship between chronic stress and oral health outcomes [26, 27].

Participants with dyed, bleached, or chemically treated hair were excluded a priori, as cosmetic hair treatments may affect measured concentrations of hair cortisol and cortisone [12]. Participants with missing data on dental examination, hair glucocorticoid measurements, or key covariates were excluded from the analytical sample. Of the 857 age-eligible participants, 307 were excluded due to missing hair glucocorticoid measurements or core covariate data, reflecting the cluster survey design in which not all registered participants completed all measurement components. Comparison of included and excluded participants revealed no differences on key variables, supporting the validity of the complete-case approach.

## Data collection

### Sociodemographic characteristics

Sociodemographic characteristics of parents were collected through structured questionnaires, including age, sex, school region, school type, ethnicity, parental marital status, parental education, and material deprivation. These covariates were selected based on prior literature and frameworks on social determinants of health, as well as their availability in the KAHS dataset, and were included as potential confounders. Ethnicity was self-reported and categorized as Kazakh, Russian, or other. Parental marital status was classified as married or unmarried (divorced, widowed, or single) [6]. Parental education was constructed as a composite variable based on the mean educational level of both parents, derived from questionnaire responses. Each parent’s education was coded ordinally and averaged to obtain a continuous parental education score. This mean score was subsequently categorized into three levels representing increasing combined parental educational attainment: low (< 1.75), medium (1.75-<2.5), and high (≥ 2.5). Material deprivation was used as a proxy indicator of household socioeconomic status and was reported in the parental questionnaire. It was assessed using three items that measured the frequency of insufficient money for food, clothing, and household bills. Responses were summed to create a deprivation index, with higher scores indicating greater economic hardship. Internal consistency of the scale was assessed using Cronbach’s alpha, demonstrating good reliability (α = 0.84). Based on the distribution of the summed score, participants were categorized into low-, moderate-, and high-deprivation groups.

### Dietary habits and oral health behaviors

Dietary habits were evaluated using parental questionnaire data on how often children consumed sugar-added foods and sugary beverage items [6]. Sugar-added foods included cakes, biscuits, chocolate, and sweets, while sugary beverages included fruit juices and carbonated soft drinks. Mean consumption scores were calculated and categorized into three levels: low (< 2.5), moderate (2.5 < 4), and high (≥ 4).

Oral health behaviors included toothbrushing frequency, dental visit patterns, and the age at which toothbrushing was initiated. Toothbrushing frequency was categorized as < 2 times per day or ≥ 2 times per day. Dental visit patterns were classified as problem-based visits or regular preventive visits. The age at which children first started brushing their teeth was categorized as before 2 years, 2–4 years, and after 4 years.

### Hair glucocorticoids

Hair cortisol and cortisone concentrations were measured in scalp hair samples collected from the posterior vertex region, which is recommended as the standard sampling site because it exhibits relatively uniform growth and lower intra-individual variability in hair cortisol compared with other scalp regions [28]. Given an average hair growth rate of approximately 1 cm per month, the proximal 3-cm segment was selected to index cumulative glucocorticoid exposure over the previous three months [29, 30]. Approximately 5 mg of hair was used for steroid extraction. Quantification of cortisol and cortisone was performed by liquid chromatography-tandem mass spectrometry (LC-MS/MS) at the Dresden Hair Cortisol Laboratory (Dresden LABservice GmbH, Germany) according to established laboratory protocols [31, 32]. Hair cortisol and cortisone concentrations were expressed as picograms per milligram of hair (pg/mg). Hair sample weight was included as a covariate in regression models to account for potential variability in analyte recovery.

### Clinical examination and dental caries assessment

Dental examinations were conducted by eight qualified dentists, with two examiners in each participating region. Prior to the start of the fieldwork, all eight dentists underwent a standardized calibration workshop based on the WHO Oral Health Surveys: Basic Methods (2013) protocol [33] to ensure consistent application of the D3 caries threshold across all participating regions. Oral examinations were performed with participants seated on school chairs under natural daylight supplemented by a dentist-mounted headlight. Disposable sterile examination kits consisting of an illuminated mouth mirror and a blunt ball-ended probe (0.5 mm tip diameter) were used. Dental caries experience was assessed using the Decayed, Missing, and Filled Teeth (DMFT) index. The maximum possible DMFT score was 28 because third molars were excluded. Caries diagnosis followed the WHO “into-dentine” threshold (D3), which records cavitated lesions extending into dentine [33, 34]. All assessments were conducted with parental consent to ensure the children’s well-being. Findings were recorded by the dentist during the examination using a standardized scoring sheet for each participant.

### Statistical analysis

Continuous variables were summarized using means and standard deviations (SD), and categorical variables using frequencies and percentages. Differences in baseline characteristics by dental caries status (DMFT = 0 vs. DMFT ≥ 1) were evaluated using chi-square tests. Descriptive analyses additionally summarized hair cortisol, cortisone, and their ratio by dental caries status, sex, and school region; medians and interquartile ranges (IQRs) were compared using nonparametric tests (Mann-Whitney U test for two-group comparisons and Kruskal-Wallis test for multi-group comparisons). Hair cortisol and cortisone concentrations were right-skewed; therefore, natural logarithmic transformations were applied. The cortisol-to-cortisone ratio was first calculated using raw hormone concentrations and then log-transformed to reduce skewness and the influence of extreme values. The log-transformed ratio was further standardized (z-score) to enable interpretation of effect estimates for a one-standard-deviation increase in glucocorticoid imbalance.

Because dental caries can be conceptualized in terms of disease presence and severity, logistic regression was used to model caries prevalence (DMFT ≥ 1), while zero-inflated negative binomial (ZINB) regression was used as a secondary analysis to examine whether the CCR association observed for caries presence extended to caries count among susceptible children. The DMFT count outcome showed overdispersion (variance 13.3 vs. mean 4.2), confirmed by a significant likelihood-ratio test for the dispersion parameter (α = 0.376, *p* < 0.001), supporting a negative binomial over Poisson specification. In addition, the distribution exhibited excess zero counts (16.3%), supporting the use of ZINB models. The count component included the same covariates as the fully adjusted logistic model. The inflation component modeled the probability of structural zeros, children with negligible susceptibility to dentine caries, and was restricted to sex, ethnicity, and school region as theoretically motivated determinants of structural zero status; this parsimonious specification was additionally informed by the limited number of zero observations (*n* = 69), which constrains reliable estimation of a more complex inflation model.

Multivariable logistic regression models were fitted to examine the association between the hair glucocorticoid ratio and the presence of dental caries (DMFT ≥ 1). Covariates were selected a priori based on their established relevance to dental caries risk and their potential to confound the association between glucocorticoid imbalance and oral health. Three sequential models were specified according to a conceptual framework. Model 1 adjusted for core sociodemographic characteristics, including sex, school region, age group, school type, and ethnicity. Model 2 additionally adjusted for socioeconomic indicators, including parental education, parental marital status, and material deprivation. Model 3 further adjusted for dietary habits and oral health behaviors, including frequency of added-sugar food consumption, sugary drink intake, toothbrushing frequency, dental visit pattern, and age at brushing initiation. All models were additionally adjusted for hair sample weight to control for laboratory measurement variability [35]. Robust standard errors were used in all models. School region was included as a fixed effect in all models to account for regional variation in caries prevalence, contextual factors, and systematic differences in dental examination by region-specific clinicians. This hierarchical modeling strategy allowed assessment of the robustness of the association across progressively comprehensive adjustment for sociodemographic, socioeconomic, and behavioral confounders. To investigate the discrepancy between unadjusted and adjusted associations, sequential isolation analyses were conducted, controlling for individual covariates one at a time and tracking the CCR estimate at each step.

Sensitivity analyses were conducted by (1) excluding implausible extreme values of the CCR, (2) comparing models using the raw log-transformed ratio versus standardized z-scores, (3) excluding hair sample weight from the fully adjusted model, and (4) applying Firth’s penalized logistic regression to address potential bias from sparse data. Results were consistent across all specifications; the cortisol ratio estimate was essentially unchanged when hair sample weight was excluded (OR = 1.36, 95% CI: 1.03–1.79 vs. OR = 1.40, 95% CI: 1.04–1.89 in the primary model), and Firth’s penalized logistic regression had a consistent estimate (OR = 1.36, 95% CI: 1.01–1.85, *p* = 0.045). Standardized models are presented as the primary option due to their improved interpretability. Fully adjusted models were additionally fitted for log-transformed hair cortisol and cortisone concentrations to examine whether associations were driven by individual glucocorticoids rather than their ratio. Multicollinearity among covariates was assessed using variance inflation factors (VIF), with all values below 5 indicating no substantial multicollinearity (Supplementary Table S6). All analyses were restricted to participants with complete data on exposures, outcomes, and covariates. Statistical significance was defined as *p* < 0.05 (two-sided). Analyses were performed using Stata version 17 (StataCorp, College Station, TX).

### Ethical considerations

The original KAHS study received approval from the National Laboratory Astana Ethics Committee [6]. Written informed parental consent and adolescent assent were obtained from all participants. The present study used a de-identified coded dataset for secondary analysis and was approved by the Ohio University Institutional Review Board under Exempt Category 4 of U.S. federal regulations. All procedures adhered to the ethical principles of the Belmont Report and the Declaration of Helsinki [36].

## Results

As shown in Table 1, among 550 participants, 462 (84.0%) had dental caries (DMFT ≥ 1), and 88 (16.0%) were caries-free. Caries prevalence did not differ significantly by sex, age group, school type, parental education, parental marital status, deprivation level, dietary factors, or oral health behaviors (all *p* > 0.05). However, significant differences were observed across school regions (*p* < 0.001), with the highest prevalence in Semey (95.5%) and the lowest in Kokshetau (72.6%), and by ethnicity (*p* = 0.011), with a higher prevalence among Kazakh children than among Russian children.

**Table 1:** Participant characteristics by dental caries status (row percentages)

Variable	Category	DMFT = 0 *n* (%)	DMFT ≥ 1 *n* (%)	*p*-value
*N*		88	462	
Sex	Boys	33 (15.1)	185 (84.8)	0.655
	Girls	55 (16.6)	277 (83.4)	
Age	14 years	54 (15.5)	294 (84.5)	0.513
	15 years	35 (17.3)	167 (82.7)	
Region	Astana	20 (22.0)	71 (78.0)	< 0.001
	Kokshetau	55 (27.4)	146 (72.6)	
	Semey	9 (4.5)	192 (95.5)	
	Oskemen	6 (10.2)	53 (89.8)	
School type	Special	56 (15.8)	298 (84.1)	0.680
	Regular	34 (17.2)	164 (82.8)	
Ethnicity	Kazakh	61 (13.7)	382 (86.2)	0.011
	Russian	21 (27.3)	56 (72.7)	
	Other	5 (17.9)	23 (82.1)	
Parental education	Not higher	22 (15.2)	123 (84.8)	0.844
	Higher	51 (15.9)	270 (84.1)	
Marital status	Not married	18 (18.8)	78 (81.3)	0.508
	Married	70 (16.0)	368 (84.0)	
Deprivation	Low	81 (16.8)	402 (83.2)	0.699
	Moderate	5 (11.9)	37 (88.1)	
	High	4 (14.8)	23 (85.2)	
Sugar foods	Low	19 (18.8)	82 (81.2)	0.729
	Moderate	33 (15.9)	175 (84.1)	
	High	34 (15.4)	187 (84.6)	
Sugary drinks	Low	70 (17.5)	330 (82.5)	0.264
	Moderate	14 (12.9)	94 (87.0)	
	High†	1 (5.9)	16 (94.1)	
Brushing	< 2/day	29 (18.8)	125 (81.2)	0.306
	≥ 2/day	58 (15.2)	323 (84.8)	
Dentist visits	Problem-based	75 (17.5)	353 (82.5)	0.114
	Preventive‡	12 (11.2)	95 (88.8)	
Brushing start	< 2 years	34 (19.9)	137 (80.1)	0.270
	2–4 years	32 (13.8)	199 (86.2)	
	> 4 years	21 (16.2)	109 (83.8)	

Percentages are row percentages within each variable category. Column totals may not sum to the header N due to missing data on individual variables

Values are n (%)

P-values from Pearson χ² tests

As shown in Table 2, median hair cortisol concentrations were 4.22 [2.31–6.28] pg/mg in children without dental caries and 4.39 [2.67–6.89] pg/mg in those with caries (*p* = 0.221). Similarly, median hair cortisone concentrations were 15.71 [10.29–21.63] pg/mg and 14.45 [10.25–22.05] pg/mg, respectively (*p* = 0.975), with no significant differences between groups. The CCR was slightly higher among children with caries but did not reach statistical significance (*p* = 0.079). In contrast, significant sex differences were observed across all biomarkers; girls exhibited higher median hair cortisol and CCR levels, but lower cortisone levels, than boys (all *p* < 0.05). Substantial regional variation was also evident (all *p* < 0.001), with the highest cortisol and cortisone concentrations observed in Oskemen and the lowest generally in Kokshetau. Glucocorticoid levels varied by sex and region but not by caries status in unadjusted analyses. (Table 2).

**Table 2:** Hair glucocorticoid biomarkers by dental caries status, sex, and school region (*n* = 550)

Biomarker	DMFT = 0 Median [IQR]	DMFT ≥ 1 Median [IQR]	*p*-value	Boys Median [IQR]	Girls Median [IQR]	*p*-value	Astana Median [IQR]	Kokshetau Median [IQR]	Semey Median [IQR]	Oskemen Median [IQR]	*p*-value
Hair cortisol (pg/mg)	4.22 [2.31–6.28]	4.39 [2.67–6.89]	0.221	3.74 [2.53–5.92]	4.68 [2.70–7.69]	< 0.001	5.02 [3.99–7.67]	3.61 [2.29–5.38]	4.03 [2.47–6.76]	7.73 [4.52–9.83]	< 0.001
Hair cortisone (pg/mg)	15.71 [10.29–21.63]	14.45 [10.25–22.05]	0.975	16.46 [12.75–24.63]	13.55 [8.51–19.60]	< 0.001	15.48 [11.55–19.68]	12.53 [9.65–17.35]	15.53 [9.28–25.31]	21.42 [14.22–30.24]	< 0.001
Hair cortisol-to-cortisone ratio	0.26 [0.20–0.34]	0.28 [0.21–0.38]	0.079	0.21 [0.17–0.26]	0.35 [0.27–0.43]	< 0.001	0.33 [0.26–0.40]	0.27 [0.20–0.36]	0.25 [0.18–0.38]	0.33 [0.25–0.41]	< 0.001

Values are medians and interquartile ranges (IQRs)

P-values for caries status and sex comparisons from Mann-Whitney U test

P-values for regional comparisons from Kruskal-Wallis test

Cortisol-to-cortisone ratio calculated as cortisol divided by cortisone concentrations (both in pg/mg); ratio is therefore dimensionless

In Model 1, adjusted for core sociodemographic factors (sex, age, region, school type, and ethnicity), a higher CCR (per SD increase) was associated with greater odds of dental caries (OR = 1.37, 95% CI: 1.07–1.74, *p* = 0.011). Compared with Astana, children from Semey (OR = 5.43, 95% CI: 1.92–15.39) and Oskemen (OR = 3.75, 95% CI: 1.21–11.67) had substantially higher odds of caries, whereas children of Russian ethnicity had lower odds than Kazakh children (OR = 0.57, 95% CI: 0.28–1.13). After adjustment for socioeconomic factors in Model 2, the association between CCR and caries remained consistent (OR = 1.34, 95% CI: 1.01–1.77, *p* = 0.040). Regional differences persisted, and moderate material deprivation showed a positive but non-significant association with caries (OR = 1.94, 95% CI: 0.57–6.60, *p* = 0.288).

In the fully adjusted Model 3, which additionally included dietary and oral health behaviors, the association between glucocorticoid imbalance and dental caries remained significant (OR = 1.40, 95% CI: 1.04–1.89, *p* = 0.027). Children from Semey (OR = 7.03, 95% CI: 1.65–29.92) continued to have higher odds of caries than those from Astana, though the wide confidence interval reflects sparse data in this subgroup and should be interpreted with caution. Russian ethnicity remained associated with lower odds of caries (OR = 0.50, 95% CI: 0.20–1.23). Moderate material deprivation showed a positive but non-significant association (OR = 3.10, 95% CI: 0.74–12.91, *p* = 0.121). None of the dietary or oral health behavioral variables reached statistical significance in the fully adjusted model. The associations between toothbrushing frequency and caries prevalence (OR = 1.29, 95% CI: 0.66–2.52, *p* = 0.456) and between preventive dental visits and caries (OR = 1.83, 95% CI: 0.86–3.88, *p* = 0.116) were not statistically significant.

Hair sample weight was not significantly associated with caries in any adjusted model. Across all three progressively adjusted models, a higher CCR was consistently associated with greater odds of dental caries (Table 3).

**Table 3:** Association between hair cortisol ratio and presence of dental caries (DMFT > 0), hair-weight adjusted

Variable	Model 1 OR (95% CI)	*p*-value	Model 2 OR (95% CI)	*p*-value	Model 3 OR (95% CI)	*p*-value
Cortisol/cortisone ratio (z-score)	1.37 (1.07–1.74)	0.011	1.34 (1.01–1.77)	0.040	1.40 (1.04–1.89)	0.027
Sex (girls vs. boys)	0.66 (0.36–1.22)	0.190	0.58 (0.29–1.15)	0.117	0.62 (0.30–1.29)	0.199
Region (Kokshetau vs. Astana)	0.73 (0.39–1.40)	0.348	0.69 (0.33–1.41)	0.306	0.65 (0.30–1.40)	0.269
Region (Semey vs. Astana)	5.43 (1.92–15.39)	0.001	4.97 (1.50-16.48)	0.009	7.03 (1.65–29.92)	0.008
Region (Oskemen vs. Astana)	3.75 (1.21–11.67)	0.022	3.17 (0.95–10.59)	0.061	2.81 (0.83–9.49)	0.096
Age (15 vs. 14)	0.87 (0.52–1.45)	0.595	0.87 (0.50–1.51)	0.613	0.91 (0.51–1.63)	0.746
School type (Regular vs. Special)	1.06 (0.58–1.94)	0.843	1.01 (0.50–2.05)	0.979	0.71 (0.33–1.52)	0.379
Ethnicity (Russian vs. Kazakh)	0.57 (0.28–1.13)	0.105	0.49 (0.22–1.07)	0.072	0.50 (0.20–1.23)	0.131
Ethnicity (Other vs. Kazakh)	1.03 (0.33–3.20)	0.953	0.88 (0.27–2.88)	0.830	0.81 (0.23–2.84)	0.738
Parental education (middle vs. higher)			1.03 (0.55–1.93)	0.916	1.01 (0.51-2.00)	0.985
Deprivation (Moderate vs. low)			1.94 (0.57–6.60)	0.288	3.10 (0.74–12.91)	0.121
Deprivation (High vs. low)			0.91 (0.11–7.39)	0.930	1.34 (0.16–11.46)	0.788
Parental marital status (unmarried vs. married)			0.92 (0.35–2.42)	0.861	1.36 (0.46–4.04)	0.581
Added-sugar foods (moderate vs. low)					1.26 (0.58–2.71)	0.562
Added-sugar foods (high vs. low)					1.30 (0.57–2.95)	0.535
Sugary drinks (moderate vs. low)					1.29 (0.61–2.72)	0.508
Sugary drinks (high vs. low)					3.35 (0.48–23.36)	0.222
Brushing ≥ 2/day (vs. < 2/day)					1.29 (0.66–2.52)	0.456
Preventive dentist visits (Regular vs. Problem-based)					1.83 (0.86–3.88)	0.116
Brushing started 2–4 yrs (vs. < 2 years)					1.47 (0.75–2.87)	0.262
Brushing started > 4 yrs (vs. < 2 years)					1.04 (0.47–2.29)	0.924
Hair weight (mg)	0.83 (0.58–1.18)	0.300	0.92 (0.61–1.39)	0.693	0.88 (0.55–1.40)	0.580

ORs for the cortisol-to-cortisone ratio represent a one standard deviation (z-score) increase. Hair sample weight was included as a continuous covariate (per 1 mg increase) to adjust for laboratory variability

Model 1: adjusted for sex, age, school region, school type, ethnicity, and hair sample weight

Model 2: additionally adjusted for parental education, material deprivation, and parental marital status

Model 3: additionally adjusted for added-sugar food consumption, sugary drink intake, toothbrushing frequency, dental visit pattern, and age at brushing initiation

All models were estimated using logistic regression with robust standard errors. The school region was included as a fixed effect in all models

Sample sizes differ across models due to missing data on socioeconomic and behavioral covariates

Wide confidence intervals for Semey and high sugary drink intake reflect sparse data in these subgroups and should be interpreted with caution

The positive association between preventive dental visits and caries prevalence likely reflects reverse causation

CCR - cortisol-to-cortisone ratio; SD - standard deviation; OR - odds ratio; CI - confidence interval

To investigate the discrepancy between unadjusted and adjusted associations, sequential isolation analyses were conducted, controlling for individual covariates one at a time. Controlling for school region alone was sufficient to reveal a significant association between CCR and caries (OR = 1.28, 95% CI: 1.03–1.58, *p* = 0.023), without adjusting for any other covariate. The estimate remained stable across all subsequent model blocks, ranging narrowly from OR = 1.28 to OR = 1.40, confirming the association is not model-dependent.

A post hoc predictive margins analysis demonstrated a graded increase in the predicted probability of dental caries with higher CCR, ranging from 74.7% (95% CI: 64.6–84.8%) at two standard deviations below the mean to 90.4% (95% CI: 85.3–95.4%) at two standard deviations above the mean, holding all other covariates at observed values (Supplementary Figure S1).

In secondary ZINB analyses, the primary objective of which was to examine whether the CCR association observed for caries presence extended to caries count among susceptible children, the CCR was not significantly associated with the number of teeth affected by dentine caries in the count component (IRR = 0.96, 95% CI: 0.89–1.04, *p* = 0.323). The CCR was included in the count component only and was not modeled in the inflation component, which was restricted to demographic and regional determinants of structural zero status. In the count component, regional differences were prominent: children from Kokshetau had significantly fewer affected teeth than those from Astana (IRR = 0.58, 95% CI: 0.45–0.75, *p* < 0.001). School type showed a borderline association (IRR = 1.17, 95% CI: 1.00-1.36, *p* = 0.050). Directionally unexpected lower counts for moderate sugar intake (IRR = 0.84, 95% CI: 0.69–1.04, *p* = 0.104) and other behavioral variables were not statistically significant. In the inflation component, which modeled the probability of being structurally caries-free, children from Semey had substantially lower odds of belonging to the always caries-free group (OR = 0.07, 95% CI: 0.008–0.43, *p* = 0.022), and Russian ethnicity was associated with higher odds of being structurally caries-free (OR = 2.85, 95% CI: 1.14–7.13, *p* = 0.025; Supplementary Table S1). The CCR was not associated with caries count in ZINB models, while being significantly associated with caries presence in logistic regression models.

Sensitivity analyses confirmed the robustness of the primary finding. Restricting the analysis to participants with optimal hair sample weight (≥ 5 mg, *n* = 342) had a consistent and significant CCR estimate (OR = 1.62, 95% CI: 1.01–2.58, *p* = 0.045; Supplementary Table S2). Firth’s penalized logistic regression, applied as a sensitivity analysis for sparse data, confirmed the CCR estimate was not materially influenced by sparse data (OR = 1.36, 95% CI: 1.01–1.85, *p* = 0.045; Supplementary Table S3). Excluding participants with implausibly extreme hormone values (*n* = 1) did not alter the association (OR = 1.95, 95% CI: 1.07–3.52, *p* = 0.028 vs. OR = 1.95, 95% CI: 1.08–3.52, *p* = 0.027 in the full sample; Supplementary Table S4). Excluding hair sample weight from the fully adjusted model had an essentially unchanged estimate (OR = 1.36, 95% CI: 1.03–1.79, *p* = 0.028), confirming the primary finding is not dependent on this adjustment. Finally, in fully adjusted models, neither log-transformed hair cortisol (OR = 1.08, 95% CI: 0.67–1.73, *p* = 0.754) nor log-transformed hair cortisone (OR = 0.73, 95% CI: 0.39–1.36, *p* = 0.319) was independently associated with dental caries prevalence, whereas the CCR remained significantly associated (Supplementary Table S5).

## Discussion

This cross-sectional study examined the association between hair glucocorticoid biomarkers and dental caries in adolescents from Kazakhstan.

### Main findings

The CCR was consistently associated with dental caries across multiple progressively adjusted models, including adjustment for sociodemographic, socioeconomic, and behavioral factors. Adjustment for sugar intake, sugary drink consumption, toothbrushing frequency, dental visits, and age at brushing initiation did not materially weaken the association, indicating that the link was not fully explained by oral health behaviors [13, 17]. The positive but non-significant associations between toothbrushing frequency and caries prevalence, and between preventive dental visits and caries, most likely reflect reverse causation rather than true behavioral effects on caries risk, whereby adolescents with existing dental disease may increase toothbrushing frequency or attend preventive visits following diagnosis or parental concern [24, 37, 38]. The fully adjusted model estimates the direct association between glucocorticoid imbalance and caries, net of behavioral factors; the contribution of any indirect pathway operating through stress-related dietary or hygiene behaviors cannot be quantified from these data and would require formal mediation analysis. The consistency of the CCR estimate across models with and without behavioral adjustment (OR = 1.37 in Model 1 vs. OR = 1.40 in Model 3) suggests that overadjustment is unlikely to have materially biased the primary finding.

These findings are broadly consistent with previous research linking cortisol and stress to dental caries experience. Abouseta et al. [13] reported significant associations between cortisol, perceived stress, and caries in adolescents and young adults, and subsequent work by the same group demonstrated relationships between glucocorticoid receptor expression in saliva and hair cortisol levels, supporting a biological link between glucocorticoid dysregulation and oral health [39]. Associations between salivary cortisol and dental caries have also been reported in children across age groups and cultural contexts [40, 41], and a literature review confirmed associations among stress, salivary cortisol, and dental caries across multiple study designs [42]. However, most prior studies used salivary cortisol, which reflects acute stress responses, rather than hair-based biomarkers that capture chronic HPA-axis activity, and none examined the CCR as a marker of glucocorticoid metabolic balance [8]. The present study contributes to this literature by examining a hair-based marker of glucocorticoid metabolic balance in relation to the prevalence of dentine caries.

The discrepancy between crude and adjusted associations reflects negative confounding by school region, as demonstrated in the sequential isolation analyses reported in the Results section. Once the regional context was controlled for, the CCR estimate remained stable across all subsequent model blocks, confirming that the association is not model-dependent. No significant association was observed between the CCR and the number of dentine lesions in ZINB models. This difference should be interpreted cautiously, as it may reflect limited statistical power, characteristics of the DMFT count distribution, or limitations of the ZINB model rather than biologically distinct mechanisms. Accordingly, the present findings should not be interpreted as definitive evidence that glucocorticoid imbalance influences susceptibility but not disease severity, and longitudinal studies with larger samples are needed to clarify this.

### Biomarker patterns

Females exhibited higher hair cortisol concentrations and higher CCR ratios than males. Similar sex differences in stress-related glucocorticoid activity have been documented in previous studies of adolescents and adults and may reflect variations in hormonal regulation, psychosocial stress exposure, or developmental factors [26, 43]. The regional variation in glucocorticoid profiles observed in this study, with the highest concentrations in Oskemen, is consistent with evidence that socioeconomic deprivation and environmental conditions are associated with elevated hair cortisol in adolescent populations [14, 44] and with findings that environmental stress factors are significantly associated with salivary cortisol levels in school-aged children [40].

### Interpretation of the cortisol-to-cortisone ratio

The CCR reflects systemic glucocorticoid balance and indicates alterations in the activity of 11β-HSD enzymes, which regulate interconversion between active cortisol and inactive cortisone and play a key role in regulating tissue exposure to glucocorticoids [3]. Although an elevated CCR may result from physiological dysregulation associated with chronic stress exposure, it should not be interpreted as a direct measure of psychological stress [3, 9, 45]. Several biological mechanisms may explain the observed association between glucocorticoid dysregulation and dental caries [13]. Activation of the HPA axis can affect immune function, inflammatory responses, and salivary composition, potentially altering the oral environment and increasing host susceptibility to cariogenic bacteria and the development of dental caries [2, 3]. This biological pathway is further supported by evidence that long-term corticosteroid use is associated with increased dental caries, consistent with the hypothesis that sustained glucocorticoid dysregulation promotes cariogenic conditions through similar mechanisms [46]. Early childhood studies have additionally demonstrated associations between salivary cortisol and caries in young children, suggesting that glucocorticoid-related effects on oral health may operate across developmental stages [41].

Chronic stress has also been associated with behavioral changes, including increased consumption of sugary foods and beverages, irregular oral hygiene, and reduced engagement in preventive health behaviors [4]. Together, these biological and behavioral pathways may contribute to increased vulnerability to dental caries in adolescents experiencing glucocorticoid dysregulation [13].

The outcome in this study was defined at the D3 dentine threshold, representing advanced cavitated lesions rather than early enamel demineralization. This distinction is relevant because dentine caries reflects a more advanced disease state associated with disruption of the oral environment, including reduction in salivary protective factors and cumulative microbial challenge, conditions that have been linked to glucocorticoid dysregulation in the literature [3, 46].

### Limitations and strengths

Several limitations should be considered when interpreting these findings. First, the cross-sectional design limits causal inference and does not allow determination of the temporal relationship between glucocorticoid imbalance and dental caries. Longitudinal studies are needed to clarify whether glucocorticoid dysregulation precedes caries development or reflects consequences of underlying behavioral and social factors. Second, the analytical sample was reduced from the original KAHS cohort, as complete dental examination data, valid hair biomarker measurements, and complete covariate information were required. Comparison of included and excluded age-eligible participants revealed no significant differences in cortisol ratio, caries prevalence, or sex distribution, suggesting selection bias is unlikely to have materially affected the primary finding.

Third, although dental examiners underwent standardized WHO-based calibration training prior to fieldwork conducted by dental epidemiologist, formal inter- and intra-examiner reliability statistics were not recorded in the original study documentation. Fourth, although dyed or chemically treated hair was an exclusion criterion, this was self-reported and may have been misclassified. Additionally, unmeasured factors such as hair growth variability and environmental contamination may have influenced cortisol and cortisone levels. Hair-based biomarkers reflect glucocorticoid exposure over the preceding weeks to months; whether a single measurement reflects a longer-standing pattern of physiological dysregulation cannot be established from a cross-sectional design. Fifth, dietary habits and oral health behaviors were self-reported and may be subject to recall or social desirability bias. Sixth, adjustment for oral health behaviors may have resulted in partial overadjustment if these factors functioned as mediators rather than confounders. However, the CCR estimate was stable across models with and without behavioral adjustment, suggesting overadjustment is unlikely to have materially biased the primary finding. Finally, residual confounding from unmeasured factors, such as exposure to psychosocial stress, access to dental services, water fluoridation, and oral microbiota composition, cannot be ruled out.

Despite these limitations, this study has several methodological strengths. First, the use of hair cortisol and cortisone measurements enabled assessment of long-term glucocorticoid exposure, providing advantages over short-term biomarkers such as salivary cortisol, which reflect acute fluctuations [15]. Second, standardization of the log-transformed CCR enabled clearer interpretation of effect sizes across models. Third, hierarchical modeling aligned with the conceptual pathways linking sociodemographic, behavioral, and biological determinants of oral health [14, 15]. Fourth, the school region was included as a fixed effect in all models to account for regional variation in caries prevalence and examiner effects, providing more reliable inference than cluster-robust standard errors with only four clusters. Finally, Firth’s penalized logistic regression confirmed the primary estimate was not materially influenced by sparse data, and consistency of results across multiple sensitivity analyses supports the robustness of the primary finding within the constraints of the study design.

### Future directions

Future longitudinal studies are needed to clarify the temporal relationship between glucocorticoid imbalance and the development of dental caries. Research incorporating repeated biomarker measurements could help capture dynamic patterns of glucocorticoid exposure across adolescence. Integrating objective behavioral assessments, psychosocial stress measures, and microbiological indicators may further improve understanding of the biological and behavioral pathways linking glucocorticoid dysregulation and oral health. Studies conducted in diverse geographic and socioeconomic contexts are also needed to assess the generalizability of these findings.

## Conclusions

In this cross-sectional sample of adolescents, a higher hair CCR was associated with greater odds of dental caries after adjustment for sociodemographic, socioeconomic, and behavioral factors. Glucocorticoid imbalance was not significantly associated with caries severity in ZINB models, though this should be interpreted cautiously given potential power limitations. Although elevated CCR may reflect physiological dysregulation associated with chronic stress exposure, it should not be interpreted as a direct measure of psychological stress, and the cross-sectional design precludes causal inference. These findings are exploratory and require confirmation in longitudinal studies before conclusions about the association between glucocorticoid imbalance and caries susceptibility can be drawn.

## Supplementary Information

Supplementary Material 1.

Supplementary Material 2.

## Data Availability

The data included in this manuscript are available from the corresponding author upon reasonable request.

## Abbreviations

DMFT: Decayed, Missing, Filled, Teeth
CCR: Cortisol-to-Cortisone Ratio
ZINB: Zero-Inflated Negative Binomial
HPA: Hypothalamic-Pituitary-Adrenal
OR: Odds Ratio
IRR: Incidence Rate Ratio
CI: Confidence Interval
SD: Standard Deviation
11β-HSD: 11β-hydroxysteroid dehydrogenase
KAHS: Kazakhstan Adolescent Health Study
WHO: World Health Organization
HBSC: Health Behavior in School-aged Children
CDHS: Children’s Dental Health Survey
NIS: Nazarbayev Intellectual School
VIF: variance inflation factors
