# Supplementary material for: Hair cortisol-to-cortisone ratio and dental caries in adolescents: a cross-sectional analysis of glucocorticoid imbalance and caries prevalence
Source: BMC Oral Health. 2026 Jun 5;26:1407. doi: 10.1186/s12903-026-08785-7 (PMC13459202; doi:10.1186/s12903-026-08785-7)
Supplement: Supplementary file 1 [file 12903_2026_8785_MOESM1_ESM.docx]

**Supplementary Tables**

**Supplementary Table S1** Zero-inflated negative binomial regression of dentine caries severity

| **Variable** | **Category** | **Count IRR** | **95% CI** | **p-value** | **Inflate OR** | **95% CI** | **p-value** |
| --- | --- | --- | --- | --- | --- | --- | --- |
| **CCR (z-score)** | Per SD increase | 0.96 | 0.89-1.04 | 0.323 |  |  |  |
| Sex | Girls vs boys | 0.97 | 0.81-1.16 | 0.731 | 1.05 | 0.37-2.75 | 0.929 |
| Region | Kokshetau vs Astana | 0.58 | 0.45-0.75 | <0.001 | 1.00 | 0.24-4.24 | 0.994 |
|  | Semey vs Astana | 1.20 | 0.93-1.54 | 0.154 | 0.07 | 0.008-0.43 | 0.022 |
|  | Oskemen vs Astana | 1.07 | 0.81-1.41 | 0.627 | 0.26 | 0.07-1.37 | 0.113 |
| Age | 15 vs 14 years | 0.95 | 0.82-1.10 | 0.489 |  |  |  |
| School type | Regular vs special | 1.17 | 1.00-1.36 | 0.050 |  |  |  |
| Ethnicity | Russian vs Kazakh | 0.98 | 0.79-1.21 | 0.839 | 2.85 | 1.14-7.13 | 0.025 |
|  | Other vs Kazakh | 0.76 | 0.54-1.07 | 0.111 | 1.26 | 0.34-4.66 | 0.778 |
| Deprivation | Moderate vs low | 1.09 | 0.77-1.56 | 0.621 |  |  |  |
|  | High vs low | 0.99 | 0.70-1.41 | 0.970 |  |  |  |
| Parental education | Middle vs higher | 0.89 | 0.77-1.03 | 0.130 |  |  |  |
| Marital status | Married vs unmarried | 1.20 | 0.90-1.58 | 0.209 |  |  |  |
| Sugar foods | Moderate vs low | 0.84 | 0.69-1.04 | 0.104 |  |  |  |
|  | High vs low | 0.85 | 0.68-1.05 | 0.123 |  |  |  |
| Sugary drinks | Moderate vs low | 0.97 | 0.82-1.15 | 0.738 |  |  |  |
|  | High vs low | 0.82 | 0.61-1.10 | 0.184 |  |  |  |
| Brushing | ≥2/day vs <2/day | 1.10 | 0.93-1.29 | 0.273 |  |  |  |
| Dentist visits | Preventive vs problem-based | 1.14 | 0.94-1.37 | 0.182 |  |  |  |
| Brushing start | 2–4 years vs <2 years | 1.10 | 0.92-1.33 | 0.295 |  |  |  |
|  | >4 years vs <2 years | 0.99 | 0.81-1.20 | 0.906 |  |  |  |
| Hair weight | Per mg | 1.05 | 0.99-1.11 | 0.123 |  |  |  |

IRR - incidence rate ratio; OR - odds ratio; CI - confidence interval; CCR - cortisol-to-cortisone ratio; SD - standard deviation.

The count component models the number of dentine-carious teeth among susceptible children. The inflation component models the probability of being a structural zero, a child with negligible susceptibility to dentine caries.

The CCR was included in the count component only. The inflation component was restricted to sex, ethnicity, and school region.

Robust standard errors used throughout.

Overdispersion confirmed by likelihood ratio test (α=0.376, p<0.001).

**Supplementary Table S2.** Sensitivity analysis restricted to participants with hair sample weight ≥5mg (n=342): association between the cortisol-to-cortisone ratio and dental caries prevalence (DMFT ≥ 1) from the fully adjusted logistic regression model.

| **Variable** | **Category** | **OR** | **95% CI** | **p-value** |
| --- | --- | --- | --- | --- |
| **CCR (z-score)** | Per SD increase | 1.62 | 1.01-2.58 | 0.045 |
| Sex | Girls vs boys | 0.45 | 0.19-1.04 | 0.063 |
| Region | Kokshetau vs Astana | 0.67 | 0.30-1.52 | 0.339 |
|  | Semey vs Astana | 5.03 | 1.14-22.30 | 0.033 |
|  | Oskemen vs Astana | 2.59 | 0.76-8.87 | 0.129 |
| Age | 15 vs 14 years | 0.73 | 0.39-1.37 | 0.326 |
| School type | Regular vs special | 0.99 | 0.44-2.26 | 0.982 |
| Ethnicity | Russian vs Kazakh | 0.51 | 0.18-1.40 | 0.190 |
|  | Other vs Kazakh | 0.41 | 0.12-1.39 | 0.153 |
| Parental education | Middle vs higher | 0.90 | 0.44-1.86 | 0.783 |
| Deprivation | Moderate vs low | 4.14 | 0.49-35.27 | 0.194 |
|  | High vs low | 0.78 | 0.05-12.16 | 0.859 |
| Marital status | Married vs unmarried | 0.75 | 0.15-3.71 | 0.724 |
| Sugar foods | Moderate vs low | 1.38 | 0.61-3.10 | 0.442 |
|  | High vs low | 1.51 | 0.62-3.68 | 0.363 |
| Sugary drinks | Moderate vs low | 1.68 | 0.73-3.85 | 0.223 |
|  | High vs low | 2.42 | 0.27-21.69 | 0.430 |
| Brushing | ≥2/day vs <2/day | 1.25 | 0.64-2.44 | 0.513 |
| Dentist visits | Preventive vs problem-based | 2.95 | 1.26-6.90 | 0.013 |
| Brushing start | 2–4 years vs <2 years | 1.74 | 0.83-3.64 | 0.141 |
|  | >4 years vs <2 years | 1.08 | 0.46-2.56 | 0.856 |
| Hair weight | Omitted |  |  |  |

OR -odds ratio; CI - confidence interval; CCR - cortisol-to-cortisone ratio; SD - standard deviation.

Robust standard errors used throughout. School region included as fixed effect.

Hair sample weight was omitted from this model due to collinearity arising from restriction to participants with adequate sample weight.

**Supplementary Table S3.** Sensitivity analysis using Firth's penalized logistic regression: fully adjusted model for dental caries prevalence (DMFT ≥ 1), n=434.

| **Variable** | **Category** | **OR** | **95% CI** | **p-value** |
| --- | --- | --- | --- | --- |
| **CCR (z-score)** | Per SD increase | 1.36 | 1.01–1.85 | 0.045 |
| Sex | Girls vs boys | 0.65 | 0.32–1.31 | 0.225 |
| Region | Kokshetau vs Astana | 0.68 | 0.32–1.44 | 0.316 |
|  | Semey vs Astana | 5.98 | 1.89–18.89 | 0.002 |
|  | Oskemen vs Astana | 2.45 | 0.78–7.70 | 0.124 |
| Age | 15 vs 14 years | 0.91 | 0.51–1.62 | 0.755 |
| School type | Regular vs special | 0.74 | 0.36–1.51 | 0.404 |
| Ethnicity | Russian vs Kazakh | 0.52 | 0.23–1.18 | 0.118 |
|  | Other vs Kazakh | 0.77 | 0.24–2.41 | 0.651 |
| Parental education | Middle vs higher | 1.00 | 0.49–2.04 | 0.993 |
| Deprivation | Moderate vs low | 2.43 | 0.57–10.37 | 0.229 |
|  | High vs low | 1.05 | 0.11–10.22 | 0.967 |
| Marital status | Married vs unmarried | 1.36 | 0.42–4.37 | 0.607 |
| Sugar foods | Moderate vs low | 1.26 | 0.59–2.68 | 0.553 |
|  | High vs low | 1.29 | 0.59–2.79 | 0.522 |
| Sugary drinks | Moderate vs low | 1.24 | 0.57–2.66 | 0.588 |
|  | High vs low | 2.19 | 0.36–13.22 | 0.394 |
| Brushing | ≥2/day vs <2/day | 1.27 | 0.68–2.38 | 0.448 |
| Dentist visits | Preventive vs problem-based | 1.71 | 0.81–3.62 | 0.157 |
| Brushing start | 2–4 years vs <2 years | 1.42 | 0.75–2.68 | 0.280 |
|  | >4 years vs <2 years | 1.01 | 0.48–2.13 | 0.980 |
| Hair weight | Per mg | 0.92 | 0.59–1.42 | 0.698 |

OR - odds ratio; CI - confidence interval; CCR - cortisol-to-cortisone ratio; SD - standard deviation.

Firth's penalized logistic regression applied as sensitivity analysis to address potential bias from sparse data. ORs derived by exponentiating penalized coefficients.

School region included as fixed effect.

**Supplementary Table S4.** Sensitivity analysis excluding participants with implausibly extreme hormone values: association between log-transformed cortisol-to-cortisone ratio and dental caries prevalence (DMFT ≥ 1).

| **Model** | **n** | **OR** | **95% CI** | **p-value** |
| --- | --- | --- | --- | --- |
| Full sample | 434 | 1.95 | 1.08-3.52 | 0.027 |
| Excluding extreme values | 433 | 1.95 | 1.07-3.52 | 0.028 |

Both models fully adjusted for sex, school region, age, school type, ethnicity, parental education, marital status, deprivation, dietary habits, oral health behaviors, and hair sample weight.

**Supplementary Table S5.** Associations of individual hair glucocorticoids with dental caries prevalence (DMFT ≥ 1): fully adjusted logistic regression models.

| **Exposure** | **n** | **OR** | **95% CI** | **p-value** |
| --- | --- | --- | --- | --- |
| Hair cortisol, ln(pg/mg) | 434 | 1.08 | 0.67-1.73 | 0.754 |
| Hair cortisone, ln(pg/mg) | 437 | 0.73 | 0.39-1.36 | 0.319 |

Both models fully adjusted for sex, school region, age, school type, ethnicity, parental education, marital status, deprivation, dietary habits, oral health behaviours, and hair sample weight.

**Supplementary Table S6.** Variance inflation factors (VIF) for the fully adjusted logistic regression model (Model 3).

| **Variable** | **VIF** |
| --- | --- |
| Cortisol/cortisone ratio (z-score) | 1.41 |
| Sex | 1.59 |
| School region: Kokshetau | 2.38 |
| School region: Semey | 2.97 |
| School region: Oskemen | 1.59 |
| Age group | 1.09 |
| School type | 1.38 |
| Ethnicity: Russian | 1.19 |
| Ethnicity: Other | 1.15 |
| Parental education | 1.3 |
| Deprivation: Moderate | 1.09 |
| Deprivation: High | 1.09 |
| Parental marital status | 1.05 |
| Added-sugar foods: Moderate | 2.1 |
| Added-sugar foods: High | 2.19 |
| Sugary drinks: Moderate | 1.1 |
| Sugary drinks: High | 1.15 |
| Brushing ≥2/day | 1.17 |
| Preventive dental visits | 1.12 |
| Brushing started 2-4 years | 1.41 |
| Brushing started >4 years | 1.43 |
| Hair sample weight | 1.43 |
| Mean VIF | 1.47 |

VIF - variance inflation factor. Values below 5 indicate no substantial multicollinearity. Mean VIF of 1.47 confirms absence of multicollinearity among covariates in the fully adjusted model. VIF calculated using linear probability model with identical covariates to the primary logistic regression model.
