# Supplementary material for: Hair cortisol-to-cortisone ratio and dental caries in adolescents: a cross-sectional analysis of glucocorticoid imbalance and caries prevalence
Source: BMC Oral Health. 2026 Jun 5;26:1407. doi: 10.1186/s12903-026-08785-7 (PMC13459202; doi:10.1186/s12903-026-08785-7)
Supplement: Supplementary file 2 [file 12903_2026_8785_MOESM2_ESM.docx]

**Supplementary Figure S1.** Predicted probability of dental caries (DMFT ≥ 1) across values of the standardized log cortisol-to-cortisone ratio (z-score), estimated from the fully adjusted logistic regression model (Model 3) using predictive margins. Predicted probabilities ranged from 74.7% (95% CI: 64.6-84.8%) at z = −2 to 90.4% (95% CI: 85.3-95.4%) at z = +2, with all other covariates held at observed values. Error bars represent 95% confidence intervals. n=434.
